# Short-Term Evaluation of Prosthetic Rehabilitation of Thin Wiry Ridge by Ridge Splitting and Simultaneous Implants Placement: Non-randomized Control Trial

**DOI:** 10.1055/s-0041-1736292

**Published:** 2021-12-04

**Authors:** Mohamed Y. Sharaf, Asharf Email Eskander, Ahmed Ibrahim Elbakery

**Affiliations:** 1 Department of Prosthodontics, Faculty of Dentistry, University of Menoufia, Menoufia, Egypt; 2 Department of Prosthodontics, Faculty of Dentistry, University of Cairo, Cairo, Egypt; 3 Department of Prosthodontics, Faculty of Dentistry, University of Ahram Canadian, Cairo, Egypt

**Keywords:** implant, knife-edge ridge, ridge expansion, piezoelectric, implant-supported prosthesis, prosthetic complications

## Abstract

**Objective** This article evaluates the success of prosthetic rehabilitation of thin wiry ridge and implants placed simultaneously in splitted ridge both clinically and radiographically.

**Materials and Methods** Twenty-one participants were enrolled of which 13 patients (8 females and 5 males) were suffering from maxillary ridge atrophy and 8 patients (5 females and 3 males) had mandibular ridge atrophy; a total of 42 implants were performed using the ridge expansion technique. The expansion was performed using the conventional disk technique, piezoelectric corticotomy, and self-threading expanders. Implants were placed and loaded with fixed partial denture after 4 months for the mandible and 6 months for the maxilla. Implant stability quotient (ISQ) was measured at T0 (implant placement) and TL (loading). Crestal bone levels were measured at different times: T0, TL, and T12 (12 months). Evaluation of prosthetic and surgical complications was carried out. Data were analyzed and compared using analysis of variance and paired *t* -tests at a significance level of 5%.

**Results** All implants met the criteria for success. All implants showed a higher mean bone loss from T0 to TL (1.259 ± 0.3020) than from TL to T12 (0.505 ± 0.163) with a statistically significant difference ( *p* < 0.0001). ISQ values sharply increased at the time of loading (72.52 ± 2.734) than at implant insertion (44.5 ± 4.062) with a significant difference ( *p* < 0.0001). Minor prosthetic and surgical complications were reported.

**Conclusion** The results from this study support the efficacy of prosthetic rehabilitation of thin wiry ridge using split ridge technique and the success of implants placed simultaneously in splitted ridge.

## Introduction

Prosthetic rehabilitation of maxillary and mandibular thin partially edentulous ridge areas represents a challenging procedure that is difficult to be restored by removable prosthesis, tooth-supported fixed partial dentures, and implant-supported prosthesis. Increasing the ridge width could be of value in improving prosthetic rehabilitation. Ridge expansion techniques of thin ridges were used as a form of preprosthetic surgery for improving the support of partial and complete dentures. However, with the introduction of osseointegration concepts and implants, ridge expansion techniques became highly recommended. 1

In general, in order to ensure a successful outcome of implants, a minimum thickness of 1 to 1.5 mm of bone should be present on both buccal and lingual/palatal aspects of the implant(s), that is, a minimum of 6 to 7 mm bone width is required for placement of an implant with a diameter of 3.5 to 4 mm. 2 3 4 Narrow alveolar ridges remain a severe challenge for placement of implants using the prosthetic-driven concept rather than bone-driven one for successful prosthetic rehabilitation regarding both the function and esthetics. 5 6

Several approved techniques were introduced to overcome narrow ridge width, including onlay bone grafts, horizontal guided bone regeneration, alveolar ridge expansion, and alveolar ridge splitting of the edentulous ridge. 2 7 8 The principal disadvantage of onlay bone grafts is the invasiveness because of the technique of bone harvesting from intraoral or extraoral sites, which increase the morbidity with the risk of bone graft resorption. 9 10 The problems of normal guided bone regeneration include the risk of membrane exposure, infection, and unpredictable rate of bone resorption after the reconstructive, regenerative procedures and implant placement. 11 12 Also, the alveolar ridge expansion technique provides a gradual increase of the ridge width and allows positioning of implants simultaneously, thus significantly reducing treatment time. However, it is recommended only for soft bone quality (D3 and D4). 13 14 15 16 Alveolar ridge splitting/expansion technique (ARST) involves splitting the alveolar ridge vertically with displacing buccal and lingual or palatal plates both in the maxilla and the mandible, creating a middle gap, usually occupied mostly by the inserted implants. 2 17 18 ARST with simultaneous placement of the dental implants arose a great interest in the last years because of the reduction of morbidity (no bone harvesting, no risk of membrane exposure, no risk of graft loss) and decreasing the postoperative swelling and pain, increasing the patient cooperation for the surgery, eliminating the need for a second surgical site as well as a second surgery, reduce the treatment cost, and reduce the total treatment time. 2 19 20

Several materials are used for prosthetic part construction. Trilor disk is one of the computer-aided design and computer-aided manufacturing systems. It is a fiberglass disc with a unique weave and epoxy resin that offers high performance. Trilor (fiber-reinforced composites) is a new technopolymer consisting of a thermo-hardening resin and a multidirectional reinforced with multidirectional fiberglass, which is used in racing cars, airplanes, and many other fields where the demand for high toughness, low weight, and excellent resistance to deformation are essential needs. Trilor is characterized by flexural resistance of 540 MPa, flexural modulus 26 GPa, and density 1.8 g/cm ^3^ . 21

This study aimed to evaluate the success of the prosthetic rehabilitation of thin wiry ridge and evaluate implants placed simultaneously in splitted ridge both clinically and radiographically.

## Material and Methods

### Patient Selection

**Fig. 1:**
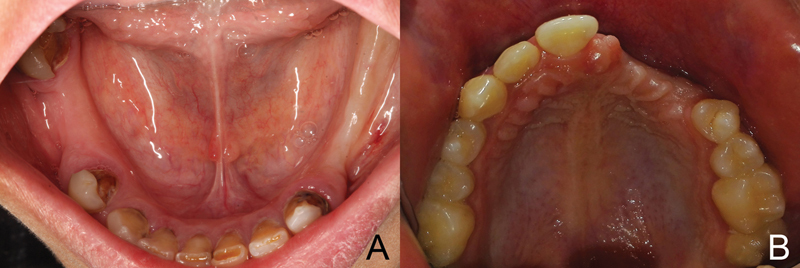
Preoperative view of both the maxilla and mandible.

Twenty-one participants were enrolled of which 13 patients (8 females and 5 males) were suffering from maxillary ridge atrophy and 8 patients (5 females and 3males) had mandibular ridge atrophy with an average age of 20 to 45 years ( Fig. 1 ) according to the following criteria: participants with a partially edentulous ridge of 2 to 4 mm of initial alveolar crest width and sufficient height of at least 8 mm from the crest of the alveolar ridge to the vital neighboring structures and good oral hygiene. In contrast, the exclusion criteria were patients who smoke more than 10 cigarettes per day and patients with any systemic disease which directly affects the bone metabolism and healing such as diabetes mellitus, osteoporosis, and periodontal disease. All included participants agreed to have the treatment and signed the informed consent. The study was approved by the Ethical Committee and adhered to the principles of the Declaration of Helsinki.

**Fig. 2:**
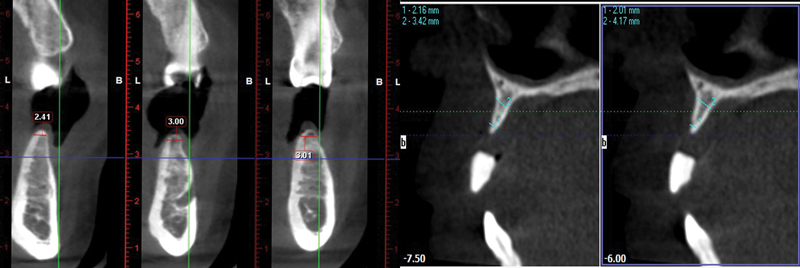
Cross-section of the proposed implant site.

Cone beam computed tomography was carried out for each patient to check the bone width, available bone height, and determine the proposed implant site ( Fig. 2 ). Alginate impressions were made, and a diagnostic wax-up was made on the study cast to fabricate a vacuum stent to locate the proposed osteotomy sites during surgery.

### Surgical Procedures

The surgical guide was disinfected by immersing it in 2% glutaraldehyde solution for 15 minutes. Patients received amoxicillin 2 g two hours before surgery. Local anesthesia was given in the proposed implant sites. The surgical guide was placed, and an explorer was used to mark the proposed implant sites. A lingual or palatal incision was made extending 6 to 8 mm mesial and distal beyond the marked implant site. The incision may extend to include the interdental papilla for the adjacent natural teeth in some cases, and a vertical incision may be made according to each case. A split-thickness flap was reflected. The surgical guide was placed again, and a round surgical bur was used to mark the proposed sites for implant placement on the bony ridge.

**Fig. 3:**
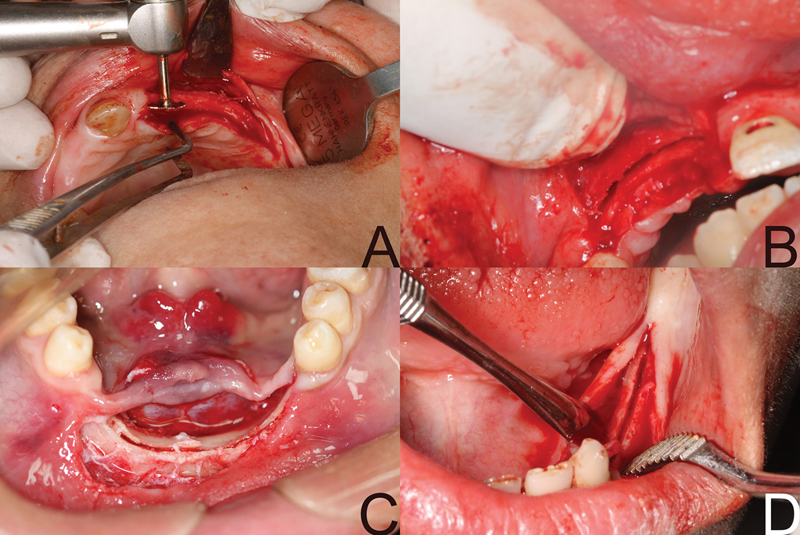
The splitted ridge.

The crestal osteotomy was made using the disk saw kit (small disk, large disk, mandrel and expanders, and finger ratchet) (Precision Dental). The small disk (6 mm diameter) was mounted within a straight handpiece and held perpendicular to the ridge, and rotated under a copious amount of coolant for making the initial osteotomy of 3-mm depth; then, the disk was replaced with larger disks 10.14 mm ( Fig. 3 ). Ultrasonic flat chisel was used to cut the area adjacent to the natural teeth and deepen the sagittal osteotomy as it should extend 5 to 7 mm in depth and 5 mm beyond the proposed implant site and just away from the adjacent natural tooth by 1 mm.

**Fig. 4:**
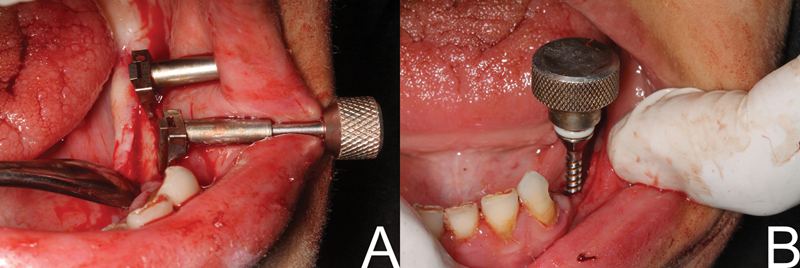
( **A** ) Use of extension crest device. ( **B** ) Use ridge expanders.

Once the osteotomies were completed, one or more extension crest devices (ECDs) (ECD consists of two surgical steel arms hinged and a transversal screw which allows a progressive activation of the device). Every complete screw turn corresponds to an activation of 0.5 mm. The maximum expansion obtainable with extension crests is 5 mm (Precision Dental), and were placed through the crestal osteotomy between the buccal and palatal/lingual plates according to the extension of the osteotomy and bone density ( Fig. 4A ).

**Fig. 5:**
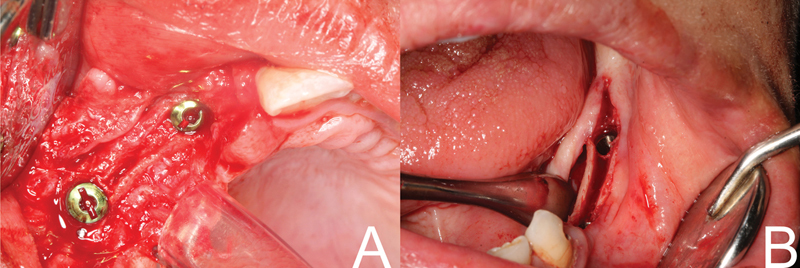
(A) Implants are flushed with crest. ( **B** ) Implants are placed subcrestal.

After each turn, a periodic pause allows the viscoelastic bone to adapt to the expansion to avoid fracture of the thin buccal plate of bone where the number of activation cycles is correlated to both bone density and surgical needs. After the required alveolar crest expansion is obtained, basal bone drilling was performed by the pilot drill (a double-level implant site preparation was performed at the basal bone level). The screw expanders (Ridge expander kit, Dentium Co.) of sequential diameters (2.3, 2.8, and 3.4 mm) were mounted into the finger ratchet and introduced to expand their corresponding future implant site slowly as every 2-mm inserted of expander drill was usually followed by 15 to 20 seconds of periodic pause giving the bone sufficient time for gradual expansion, and then replaced with successive expanders of a larger diameter till the proposed final implant size was achieved ( Fig. 4B ). As the last expander was removed, the implant was placed immediately to prevent the collapse of the expanded bone. The implant was installed in the osteotomy site and rotated gradually subcrestal as much as we can or flushing with the bone ( Fig. 5 ) (insertion torque 25 N/cm ^2^ ), then another implant was placed parallel to the first one. The flap was repositioned and sutured.

Postoperative care included cold packs applied for 20 minutes every hour for 6 hours postoperatively. The patient was kept on a soft diet for the first 48 hours. The patient was advised to rinse with chlorhexidine 0.12% twice a day for 10 days till suture removal. At least 4 months were needed for bone healing in the mandible and 6 months for the maxillary arch.

### Prosthetic Procedures

Removable partial dentures were not allowed to be used during the first month in the treated areas. 19 All implants were loaded with a fixed prosthesis (cement-retained). Healing abutments were placed, and the soft tissue was allowed to heal for 1 week. Healing abutments were replaced by the impression transfer (closed tray technique), and the final impression was made using a rubber base (ZetaPlus, Zhermack SpA). The impression transfers were unscrewed from the implants and connected to the implant analog, and placed within the impression. Tissue mimic material was applied and the cast was poured by extra hard stone. The final abutment replaced the impression transfer.

**Fig. 6:**
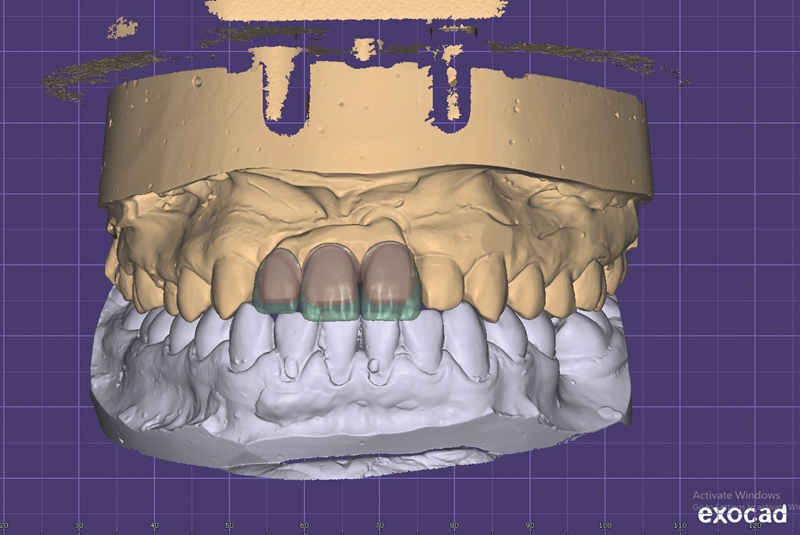
Designing of the Trilor fixed partial denture.

**Fig. 7:**
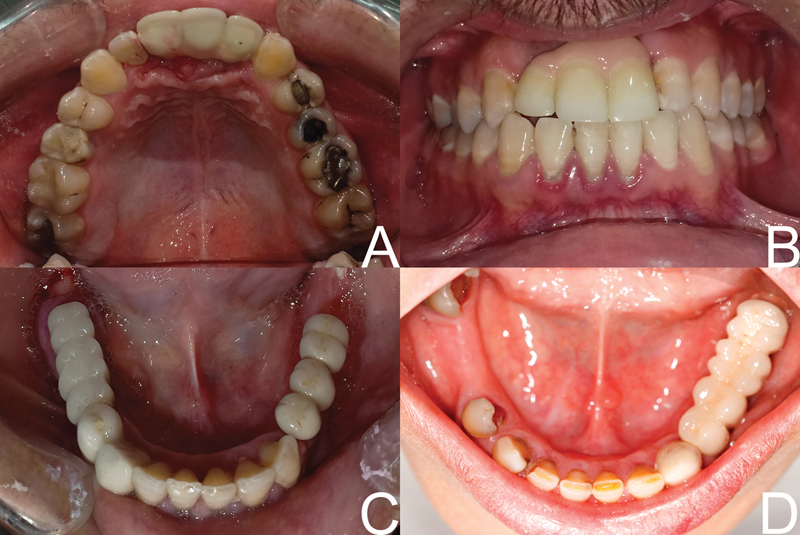
Final prosthesis cemented intraoral.

For Trilor fixed partial denture, the cast was scanned by a laboratory scanner to obtain a Standard Tessellation Language file. Design of fixed partial denture was carried out on dental software (Exocad, Darmstadt, Germany) ( Fig. 6 ). After finishing the design, the framework was printed by a three-dimensional printer (Mogassam Co. LLC) and tried intraoral for accuracy, adaptation, marginal fit, and esthetics. The framework was milled from a Trilor disk of 98.5*23 mm (Trilor, Bioloren), and veneering material was made from visio.lign and crea.lign (Bredent UK) and tried intraoral for any modifications. Cementation of the final prosthesis was done using temporary cement (Prevest Denpro Zinconol Dental Cement) for 2 weeks and later the prosthesis was removed and the abutment screw retightened. Finally, the prosthesis was cemented by glass ionomer (Medicem Promedica Dental Material GmbH) ( Fig. 7A , 7B ).

For porcelain fused to metal (PFM) fixed partial denture wax pattern was carried out. Investing and casting of the wax pattern was done. The metal framework of the fixed partial denture was checked for accuracy, adaptation, and marginal fit, then the firing of the porcelain was done. Cementation of the final prosthesis was done using temporary cement (Prevest Denpro Zinconol Dental Cement) for 2 weeks and later the prosthesis was removed and the abutment screw retightened. Finally, the prosthesis is cemented by glass ionomer (Medicem Promedica Dental Material GmbH) ( Fig. 7C , 7D ).

### Evaluations

Implant success rate involving the following clinical parameters for success was suggested by Albrektsson et al. 22

Implant stability quotient (ISQ) was assessed by resonance frequency analysis (RFA) using the Osstell apparatus (Osstell; Osstell AB). SmartPegs were attached to the implants. For each implant, four readings were taken at the buccal, lingual, mesial, and distal sides at the time of implant placement and loading. The average ISQ values were calculated for all surfaces. 23 24

*Radiographicexamination* : For ensuring standardization of measurements, digital radiographs were taken using a long-cone paralleling technique at the time of implant insertion (T0), loading (TL), and 12 months (T12) after prosthesis insertion. For each image, the distance from the implant platform (A point) to the crestal bone level (B point) was calculated (in mm) using the measuring tool of the software to indicate the vertical bone level (X) in mm. X measurements were calibrated based on the known implant length to detect magnification errors. Alveolar bone loss (ABL) was calculated by subtracting X at TL and T12 from X at T0 and TL, respectively. ABL value was measured at the mesial and distal surfaces of each implant, and the mean value was calculated.

*Prosthetic evaluation* : During follow-up for 1 year, the status of the prostheses was screened for the presence of any complication (abutment screw loosening, abutment screw fracture, fracture of veneer material, or loss of retention and decementation).

## Results

The data were collected for all participants during follow-up with no dropout. All implants ( Table 1 ) met the modified Albrektsson et al criteria for success. 22

**Table 1:** Implant dimension and position

	3.6*10	3.6*8	Total
Maxillary	23	3	26
Mandibular	16	−	16
Total	39	3	42

*Implants stability assessment* : There was an increase in ISQ from implant placement till implant loading for all implants with statistically significant difference ( *p* < 0.0001) between values at insertion (44.5 ± 4.062) and loading (72.52 ± 2.734) as well as a statistically significant increase ( *p* < 0.0001, *p* < .0017) was observed in ISQ at insertion and loading for anterior implants in the mandible (49 ± 2.7688, 74.83 ± 1.95) than anterior implants in the maxilla (40.75 ± 2.358, 71.25 ± 2.106), respectively ( Tables 2 and 3 ). A statistically significant increase ( *p* < 0.0047, *p* < 0.035) was observed in ISQ at insertion and loading for posterior implants in the mandible (47.7 ± 1.552, 74.1 ± 2.7 73) than posterior implants in the maxilla (44.6 ± 2.615, 71.6 ± 2.107), respectively ( Tables 2 and 3 ), and also a statistically significant increase ( *p* < 0.0001, *p* < 0.0002) was observed in ISQ at insertion and loading for implants in the mandible (48.18 ± 2.185, 74.37 ± 2.521) than implants in the maxilla (42.23 ± 3.0922, 71.38 ± 2.113), respectively ( Tables 2 and 3 ). There is a statistical significance difference ( *p* < 0.0087) between anterior implants (43 ± 4.431) and posterior implants (46.15 ± 2.650) at insertion ( Tables 2 and 3 ). A statistically significant increase ( *p* < 0.0087, *p* < 0.0266) was observed in ISQ at insertion for posterior implants (46.15 ± 2.650) than anterior implants (43 ± 4.431) as well as between implants in male (46.75 ± 4.057) and implants in female (43.88 ± 3.84), respectively ( Tables 2 and 3 ).

**Table 2:** The mean and standard deviation of Osstell reading (ISQ) at implant placement and loading

Place of implants	Anterior maxilla		Posterior maxilla		Total maxilla		Anterior mandible		Posterior mandible		Total mandible		Total anterior		Total posterior		Male		Female	
No of implants	16		10		26		6		10		6		22		20		16		26	
	Mean	SD	Mean	SD	Mean	SD	Mean	SD	Mean	SD	Mean	SD	Mean	SD	Mean	SD	Mean	SD	Mean	SD
Installation	40.75	2.3584953	44.6	2.6153394	42.23	3.0922694	49	2.7688746	47.7	1.5524175	48.18	2.1857136	43	4.4312937	46,15	2.6509432	46,75	4.057	43.88	3.84
Loading	71.25	2.106	71.6	2.107	71.38	2.113	74.83	1.95	74.1	2.773	74.37	2.521	72.22	2.609	72.85	2.761	73.125	2.77	71.73	2.272

Abbreviations: ISQ, implant stability quotient; SD, standard deviation.

a Significant at *p* ≤ 0.05.

**Table 3:** The comparison of Osstell reading (ISQ) at implant placement and loading

	Anterior maxilla vs. ant mandible				Posterior maxilla vs. post mandible				Maxilla vs. mandible				Total anterior vs. total posterior				Total implants in male vs. females			
	Difference	Standard error	95% CI	*p* -Value	Difference	Standard error	95% CI	*p* -Value	Difference	Standard error	95% CI	*p* -Value	Difference	Standard error	95% CI	*p* -Value	Difference	Standard error	95% CI	*p* -Value
Installation	8.250	1.181	10.7134 to 5.7866	0.0001 a	3.1	0.962	1.0797 to 5.1203	0.0047 a	5.950	0.885	7.7386 to 4.1614	0.0001 a	3.15	1.141	0.8436 to 5.4564	0.0087 a	2.870	1.246	5.3892 to –0.3508	0.0266+
Loading	3.580	0.990	1.5148 to 5.6452	0.0017 a	2.500	1.101	0.1878 to 4.8122	0.0356 a	2.990	0.723	1.5293 to 4.4507	0.0002 a	0.630	0.829	1.0449 to 2.3049	0.4516	1.395	0.786	2.9834 to 0.1934	0.0835

Abbreviations: CI, confidence interval; ISQ, implant stability quotient.

a Significant at *p* ≤ 0.05.

*ABL assessment* : Thirty implants were placed subosseous while 12 placed flushing with crestal bone margins. All implants showed a higher mean bone loss from T0 to TL (1.259 ± 0.3020) than from TL to T12 (0.505 ± 0.163) with a statistically significant difference ( *p* < 0.0001). A statistically significant increase ( *p* < 0.0306) in the mean of ABL was observed in anterior implants in the maxilla (0.618 ± 0.120) than anterior implants in the mandible (0.2466 ± 0.634) from TL to T12 ( Tables 4 and 5 ). A statistically significant increase ( *p* < 0.0001) in the mean of ABL was observed for implants in the maxilla (0.581 ± 0.123) than implants in the mandible (0.382 ± 0.139) from TL to T12 ( Tables 4 and 5 ). A statistically significant increase ( *p* < 0.0017) in the mean of ABL was observed for all anterior implants (1.39 ± 0.321) than all posterior implants (1.115 ± 0.13982) from T0 to TL ( Tables 4 and 5 ). A statistically significant increase ( *p* < 0.0045) in the mean of ABL was observed for implants in the female participants (0.583 ± 0.170) than male participants (0.435 ± 0.145) from TL to T12 ( Tables 4 and 5 ).

**Table 4:** The mean and standard deviation of alveolar bone loss at implant loading and after 1 year

	Anterior maxilla		Posterior maxilla		Total maxilla		Anterior mandible		Posterior mandible		Total mandible		Total anterior		Total posterior		Female		Male	
Implants no.	16		10		26		6		10		16		22		20		26		16	
	**Mean**	**SD**	**Mean**	**SD**	**Mean**	**SD**	**Mean**	**SD**	**Mean**	**SD**	**Mean**	**SD**	**Mean**	**SD**	**Mean**	**SD**	**Mean**	**SD**	**Mean**	**SD**
T0/T1	1.35625	0.351	1.105	0.169	1.2596154	0.31956077	1.4833333	0.195	1.125	0.193	1.259375	0.26053955	1.39	0.321797	1.115	0.1824	1.184	0.211	1.228	0.407
Loading	0.618125	0.120	0.541	0.0858	0.58115385	0.12336085	0.24666667	0.634	0.464	0.10403	0.3825	0.13908181	0.516	0.1975391	0.493	0.107	0.583	0.170	0.435	0.145

Abbreviation: SD, standard deviation.

a Significant at *p* ≤ 0.05.

**Table 5:** The comparison of alveolar bone loss at implant loading and after 1 year

Anterior maxilla vs. anterior mandible				Posterior maxilla vs. posterior mandible				Maxilla vs. mandible				Total anterior vs. total posterior				Male vs. female			
Difference	Standard error	95% CI	*p* -Value	Difference	Standard error	95% CI	*p* -Value	Difference	Standard error	95% CI	*p* -Value	Difference	Standard error	95% CI	*p* -Value	Difference	Standard error	95% CI	*p* -Value
0.127	0.153	0.1917 to 0.4459	0.4155	0.020	0.081	0.1504 to 0.1904	0.808	0.00	0.095	0.1921 to 0.1916	0.998	0.275	0.082	–0.4404 to –0.1096	0.0017	0.044	0.110	–0.1787 to 0.2667	0.6917
0.371	0.160	0.7046 to –0.0383	0.0306 a	0.077	0.043	0.1666 to 0.0126	0.0877	0.199	0.041	0.2818 to –0.1155	0.0001 a	0.023	0.050	0.1235 to 0.0775	0.646	0.148	0.049	–0.2474 to –0.0486	0.0045

Abbreviation: CI, confidence interval.

a Significant at *p* ≤ 0.05.

*Prosthetic complications* : Implant-supported prosthesis was involved in this study. Prosthetic complications were loosening of screw/abutment in 8 patients (38%) within the first 6 months of prosthetic service and 2 patients (9.5%) at second 6 months, while no ceramic fracture, no framework/occlusal material fracture, no screw fracture, and decementation occurred in 1 patient (4.7%).

*Surgical complications* : Buccal plates cracked at two implant sites (4.7%) in the mandible. Swelling and edema were observed postoperatively in 6 cases (28.5%) and disappeared within 3 days from surgery and finally transient paresthesia (1 case: 4.7%) and disappeared within 2 weeks.

## Discussion

ARST represents an effective and validated form of expansion technique for narrow ridges with simultaneous implants placement with a survival rate of 97.4 to 100%, and the success rates of implants are comparable to implants placed in bone without ARST which matches with our study success and survival rate. 9 23 24 25 26

Classic ridge-splitting procedures involve razor-sharp bone chisels, rotating, oscillating saws, or saw disc. The use of bone chisel traumatizes the bone and could stress the patient during surgery, it is time-consuming and require complex technical skills to be managed efficiently. Rotating and oscillating instruments are safer, less threatening for the patient, better control during cutting along a narrow alveolar ridge, and appear less traumatic to the bone. Additionally, less bone is lost because the micro-saw creates much thinner cuts than conventional burs while reducing the risk of encroaching the gingiva, the lips, or the tongue, limiting their accessibility and complicating the procedure. 6 10 16 Ultrasonic device produces easier, safer, and more precise cut. It allows curved cuts that are impossible with rotator or oscillating saws, 27 providing good visibility in the surgical field, reducing the psychological stresses on patients under local anesthesia, with no risk of injury to soft tissue, and also reduce the risk of complications in the treatment of extremely atrophic crests; however, its time consuming. 6 10 27 28 29 30 31 32 Thus, the combination of using rotating saws and piezoelectric instrument facilitates the ARST procedures and gain the advantages of both instruments.

A systematic review by Milinkovic and Cordaro and a meta-analysis by Elnayef et al evaluated the different alveolar bone augmentation procedures for implant placement. They found that the alveolar crest-split technique had minimal technical complications and a high implant survival rate. 33 34 While the main complication of ARST was reported to fracture the buccal bone, which is increased with the narrower ridge of less than 1 mm buccal and lingual cortical plates. 12 33 34 35 36 Buccal wall fracture was reported in some studies to reach up to 14.0%. 35 However, in this study, no fracture occurred, only cracks appeared at the buccal plate at two implant sites in the mandible, this could be attributed to the presence of highly dense bone and little cancellous in the arch. 37 Decreasing the complication associated with ARST may be attributed to the distribution of expansion forces by ECD, sufficient time, and multiple pauses between each turn of the expansion device to overcome the resistance during the expansion of the buccal cortical plate, thus decreasing the risk of fracture. In addition, the minimal included ridge width was 2.0 mm at the crest of the ridge. 2 35 38 A systematic review reported that the failure rate is more likely if implants are loaded within a period shorter than 3 months. 23 The dental implants in this study were loaded at 4 months for the mandible and 6 months for the maxilla. 35 Moreover, splinting of dental implants would decrease the stresses on each implant. 20 24

The use of a split-thickness flap was significant as the periosteum should not be stripped off from the buccal bone plate, affecting the blood supply and allowing rapid revascularization of the expanded bony plate. The expanded segments with elevated periosteum will undergo resorption because of the lack of nourishment, particularly for the thin buccal cortex, followed by implant thread exposure. 39 The periosteum has another function in treating the minute fractures that might occur during the splitting procedure 40 41 42 and decreasing the percentage of bone loss by 9.5% for the buccal bone plate, 7.9% for the palatal bone plate, and 3.5% for the mesiodistal bone plate as reported by Mounir et al. 43 While disadvantages of the partial flap are excessive bleeding, resulting in lousy visualization of the surgical sites. 6

The use of expanders allowed blunt lateralization of the buccal cortex during expansion, thus decreasing the risk of fractures and heating during drilling. It increases bone quality surrounding the implant due to the compression of the spongiosa at the sidewalls of the osteotomy site without any bone removal. 43 Double-level implant site preparation allowed proper primary stability with the required expansion, which was frequently difficult to obtain with traditional split crest techniques. 39

With palatal or lingual incision, the buccal plate is preserved by placing the incision to the palatal or lingual sides where the cortical plates are thicker and resistant to resorption. 39 The extended osteotomy beyond the proposed implant site allowed the plates to expand or bow during the preparation of the osteotomy preparation and implant insertion, and periodic pauses allowed the viscoelastic bone to adapt to the expansion. 6 The osteotomy gap was between 3 and 5 mm, which was left to be filled with the organized blood clot to be replaced by woven bone, allowing normal wound healing resembling an extraction socket and fracture repair that heal by secondary intension without the need for bone grafting or using guided regenerative techniques. 6 44 45

The use of RFA was beneficial in providing clinical evidence about implant-bone interface during the phases of treatment, 37 where the acceptable stability range, based on many studies made with RFA, lies between 55 and 85 ISQ with an average ISQ level of 70. 46 47 In the present study, the mean ISQ value at insertion was 44.5 ± 4.062 and loading was 72.52 ± 2.734. These results indicated a valuable improvement of the implant's stability which is the main goal for achieving successful osseointegration. The lower ISQ values measured at implant insertion explain the surrounding area where implants were placed in a gap filled with blood clot and minimal implant surface being anchored in bone; later on, a significant increase in the ISQ values was observed at 4 to 6 months, denoting the ability to load implants. 48 Several studies have demonstrated the correlation between bone quality and ISQ values, and it appears that the stiffness of the implant-bone interface increases as the peri-implant bone becomes denser during the healing and remodeling process. 33 34 35 48

The reported success rates of implants placed with ARST were comparable with those placed in bone without ARST. However, the few available and included data indicated that a slightly more pronounced marginal bone loss could be expected than implants placed in bone without ARST. 3 The crestal bone loss that occurs secondary to the ridge-splitting technique is a serious obstacle to the success of the operation and remains the challenging feature of that procedure, 43 which was reflected by the radiographic results as most of the ABL occurred at first 4 to 6 months before loading regardless the anatomical position of placed implants or the quality of bone. In this study, placing most of the implants (30 implants) subossous help decreasing the postoperative ABL as the ABL was 1.259 ± 0.3020 mm. However, since some implants were initially placed subcrestal, the net final ABL from the implant platform was 0.775 ± 0.3185 mm, where the average reported crestal bone loss was between 0.8 and 2.0 mm, and in a study by González-García et al the mean ABL was 0.542 mm. 48 49 Nevertheless, after loading, the only significant differences were between implants in the anterior maxilla and anterior mandible and between implants in the maxilla and mandible. The better quality of bone denotes less bone resorption and better implant osseointegration, as proved with implant stability by Osstell.

The main prosthetic complications of implant-supported restorations were the screw loosening, framework or occlusal material fracture, screw fracture, and decementation. 50 Although abutment screw loosening is one of the most frequent prosthetic complications that has been associated with the ARST, 34 a study by Garcez-Filho et al observed that 6 of 8 complications were due to abutment screw loosening. 51 In this study, 8 of 9 complications were due to abutment screw loosening.

In this study, using anterior fixed partial dentures of Trilor and posterior one of PFM did not affect stress distribution and stress values at the bone tissue surrounding the implant. 52 53 However, Türk et al mentioned that different materials transmit different stress to the underlying structures. 54

The main limitations of the present study are the small sample size and short follow-up duration as the study was continued for 12 months for serving as prosthetic abutments, which represent the least period to evaluate the implant success, 20 and its recommended to place grafting materials to decrease the ABL as the ABL is considered an obstacle for the success of implants in splitted ridge.

## Conclusion

This study supports the efficacy of prosthetic rehabilitation of thin wiry ridge using split ridge technique and the success of implants placed simultaneously in splitted ridge.
